# Sterile Insect Technique: Investigating the Impact of Larval Diet and Ionizing Radiation on the Mosquito Bacterial Microbiota

**DOI:** 10.3390/insects17090901

**Published:** 2026-08-27

**Authors:** Hailey A. Luker, Mya Hammock, Tathagata Debnath, Anjali Karki, Immo A. Hansen

**Affiliations:** 1 Department of Biology, New Mexico State University, Las Cruces, NM 88003, USAanjali13@nmsu.edu (A.K.); 2 Department of Computer Science, New Mexico State University, Las Cruces, NM 88003, USA; tathadbn@nmsu.edu

**Keywords:** sterile insect technique, bacterial microbiota, males, *Aedes aegypti*, larval diets

## Abstract

The Sterile Insect Technique (SIT) is a species-specific and effective insect population control technique that has the potential to be an alternative to chemical insecticides. The use of the SIT for mosquito population control is currently in development. An obstacle to the success of this technique is the decline in fitness of radiation-sterilized males. We propose that radiation exposure and specific larval diets change the bacterial microbiota of sterile adult male *Aedes aegypti*. To test this, we reared larvae on either a cat or fish food diet, irradiated the pupae, and isolated DNA from adult male abdomens three days post-irradiation. We then used 16s rRNA sequencing technology to characterize the bacterial microbiota. Our findings show for the first time in mosquitoes that larval diets change the effect of ionizing radiation on the adult bacterial microbiota.

## Simple Summary

Due to the increase in insecticide resistance in mosquitoes, there is a need for new methods to control mosquito populations. The Sterile Insect Technique (SIT) is a promising method that relies on sterilizing males using radiation. The sterile males compete with wild males to mate with wild females. The females that mate with sterile males will lay eggs that do not hatch. The goal of the SIT is to sharply reduce the mosquito population in an area. A problem with the SIT is that the sterile males are less fit than wild males. It has been shown that the bacterial microbiota plays a role in the fitness of mosquitoes. Here we investigate the impact of ionizing radiation and larval diet on the bacterial microbiota of the adult male Yellow Fever mosquito, *Aedes aegypti*. To test this, we fed larvae on either a commercially available cat or fish food diet, irradiated the pupae, and isolated DNA from adult male abdomens. We used 16s rRNA sequencing to identify the bacterial microbiota composition, abundance, and diversity in the different treatment groups. We found that larval diet impacts the adult male mosquito bacterial microbiota and alters the effect of ionizing radiation on the bacterial microbiota.

## 1. Introduction

For over a century, source reduction and insecticides have been the primary tools used for managing mosquito populations. Source reduction is a method of removing and/or preventing potential mosquito breeding sites, like containers and pots that can hold standing water, roadside ditches, and ornamental ponds [1,2,3]. The efficacy of this method can be limited, because nearly any standing water can suffice as a breeding site for some key mosquito vectors like *Aedes aegypti* [4,5,6].

Insecticides, whether larvicides or adulticides, target either the aquatic or terrestrial stage of the mosquito lifecycle. Mosquito breeding sites can be treated with bacterial larvicides, insect growth regulators, or oils and films [7,8,9,10,11]; while yards and foliage can be treated with adulticides like organophosphates, pyrethrins, and pyrethroids that are administered through coils, foggers, or yard and aerial sprays [12,13,14]. However, the effectiveness of insecticides for managing local mosquito populations has been greatly reduced due to the occurrence of high levels of insecticide resistance in all key vector mosquito species [15,16,17]. In addition, frequent reports of off-target effects and the persistence of insecticides in the environment create significant concerns associated with some chemical insecticides [18,19,20,21]. An example of this is the organochlorine pesticide, Dichlorodiphenyltrichloroethane, more commonly known as DDT [22,23,24]. DDT is a potent insecticide that was widely used in public health programs and agriculture to control insect pests. However, DDT resistance evolved quickly in many pest species, and it was later banned in many countries due to evidence of severe negative impacts to the environment and potential human health concerns [25,26,27]. Due to the above-mentioned limitations, there is a severe need for additional means to manage mosquito populations [17,28,29,30,31,32].

Integrative mosquito management (IMM) is an approach used for the sustainable management of mosquito populations [33,34]. IMM combines methods like surveillance, chemical and biological control, source reduction, and public education to limit vector mosquito populations [35,36,37]. Some novel techniques include the use of biopesticides or the release of modified mosquitoes. Biopesticides like some bacterial larvicides have been shown to have fewer off-target effects and environmental impacts compared to synthetic insecticides [38,39]. Methods that depend on the release of modified mosquitoes like the Sterile Insect Technique (SIT), *Wolbachia*-based techniques, and Release of Insects carrying a Dominant Lethal gene are highly species-specific and have little to no perceived impact on the environment [40,41]. These novel techniques can result in the decline of local mosquito populations without the use of chemical insecticides and may reduce transmission of disease-causing pathogens. However, these methods are still in development and have some obstacles that must be resolved before they can be effectively integrated into IMM strategies.

The current study is focused on an obstacle in the application of the Sterile Insect Technique (SIT). The SIT involves the sterilization and release of massive amounts of males of a specific arthropod into a region infested with that arthropod. Those sterile males will mate with their wild female counterparts resulting in a reduction in or absence of viable offspring, ultimately decreasing that arthropods’ population [42]. The first documented success of the SIT was the eradication of the New World Screwworm (NWS) (*Cochliomyia hominivorax*) from the island of Curacao in 1954 [43,44]. The success in Curacao propelled the use of the SIT for NWS in Orlando, Florida, and eventually led to the eradication of the NWS in the North and Central America stopping at Panama in 2006 [45]. While these successes demonstrate the effectiveness of this technique, the recent reemergence of the NWS in North America in 2025, demonstrates that the SIT requires continuous funding, monitoring, and adaptive strategies to avoid the resurgence of the targeted pest arthropod [46].

The SIT has been further developed as a species-specific alternative to insecticides for controlling populations of fruit flies and other agricultural and livestock pests [47,48,49]. This method has also been tested for the control of vector mosquito populations, but with varying success [50,51]. Some challenges that have emerged during the development of the SIT for vector mosquitoes are (1) the need for facilities devoted to the efficient mass rearing of mosquitoes of a specific species, (2) the equipment and dosage to completely sterilize males, (3) the resources to sex-sort the males from the females, (4) the official approval to release mosquitoes into an area, (5) the resources for the transport and sustained systematic release of sterilized males, and (6) the fitness or mating competitiveness of these sterilized males [52,53,54,55].

Male mosquitoes are often sterilized using either gamma or X-ray radiation [56]. The use of X-rays is a more recent development and is a more accessible, affordable, and safer alternative to the use of gamma rays [57]. Ionizing radiation sterilizes male mosquitoes by damaging the DNA of their spermatozoans. However, to do this, the whole mosquito is irradiated, and the DNA of somatic cells is also damaged [58,59,60]. This can result in radiation sickness, lowering male fitness and mating success. Using the lowest dose of radiation that still infers a high percentage of sterility is a way to minimize radiation sickness. However, this dosage can vary greatly depending on the species of mosquito, ranging from as low as 40 grays (Gy) to as high as 110 Gy for some *Anopheles* species [61,62,63]. Another variable to consider is the life stage of the mosquito when it is irradiated. Typically, mosquitoes are irradiated either during the late pupal stage or the early adult stage. Irradiating at the pupal stage is more time efficient and requires less effort for mosquito handling; however upon pupation, the adult males are less fit than males that were irradiated as adults [61,64,65]. To help minimize DNA damage of somatic cells caused by irradiation, some strategies have been explored such as treating larvae with hypoxia or treating mosquitoes with radioprotectants before irradiation [63,66,67].

Ionizing radiation not only damages germline and somatic cells, but it can also impact microbiome diversity and composition in animals [68,69,70]. Studies in humans have shown that altered microbiome communities can negatively affect immunity and fitness [71,72]. In mosquitoes, the microbiome has been observed to impact all facets of the life cycle, i.e., larval growth and development, adult fitness, reproduction, and even vectorial capacity [73,74,75,76,77].

Our first hypothesis is that the *Aedes aegypti* larval diet influences the adult male bacterial microbiota. Our premise is that there is transstadial transmission of some larval bacterial microbiota to the adult stage. Next, we hypothesized that ionizing radiation has an effect on the adult male *Ae. aegypti* bacterial microbiota. Lastly, we hypothesized that the bacterial microbiota of adult *Ae. aegypti* males that were reared on different larval diets will have different responses to ionizing radiation.

To investigate these hypotheses, we reared *Ae. aegypti* larvae on two different diets: cat or fish food. Late-stage pupae from these two groups were then dosed with 50 Gray (Gy) of ionizing radiation. We performed 16S rRNA sequencing on these groups and compared them to non-irradiated controls (see Graphical Abstract).

## 2. Materials and Methods

### 2.1. Mosquito Rearing

Late-stage pupae (24–36 h after pupation) and adult male *Aedes aegypti* mosquitoes from the UGAL (University of Georgia Laboratory) strain were used for this study. Approximately, 600 *Ae. aegypti* mosquitoes were hatched per ethanol-sterilized tray (L44 × W36 × H6 cm) containing 3L of deionized (DI) water. One tray of mosquitoes was hatched and used for each individual treatment and replicate. Two days after hatching, larvae were counted and transferred to additional sterilized trays in a ratio of 1 larva per 4 mL water. Larvae were fed ad libitum on one of the specified larval diets: a fish food diet (TetraMin Tropical Tablets, Blacksburg, VA, USA) or a cat food diet (Special Kitty, Walmart Stores Inc., Bentonville, AR, USA). Water was changed as needed. Pupae were stored in ethanol-sterilized dishes containing 200 mL DI water for 24 to 36 h and then used in experiments. After control or irradiation treatment, pupae were re-transferred to a sterile dish and stored in bleach-sterilized Bug Dorm cages (L7 × W7 × H7 cm Bug dorm Company, Taichung, Taiwan) with a 10% sucrose solution in autoclave-sterilized flasks. The cages were stored in an insectary room that maintained a temperature of 27 °C and 80% relative humidity with a light/dark cycle of 14/10. Once the treated and control male mosquitoes eclosed, they were immediately separated into a new cage to prevent mating with females.

### 2.2. X-Ray Irradiation

The MultiRad350 with an integrated dosimeter (Precision X-Ray Irradiation, Madison, CT, USA) was used to irradiate pupae. For both control and experimental groups, 25 pupae were transferred into Petri dishes containing 30 mL DI water. One Petri dish at a time was placed into the irradiation chamber. In experimental groups, pupae were dosed with 50 Gy of ionizing radiation, which took approximately 7 min. For control groups, the pupae were placed in the irradiation chamber for 7 min without turning on the X-ray. For simplicity, we will refer to our groups in shortform as their larval diet and irradiation status; for example, “Cat Control” refers to adult male *Ae. aegypti* that were reared on a cat food larval diet and not irradiated (see Table 1).

### 2.3. Mosquito Dissections and DNA Extraction

Subsequently, 3 days after pupal irradiation, the abdomens of 50 adult male mosquitoes were dissected per replicate. A total of four replicates for both larval diet and radiation treatment groups were collected. Mosquitoes were anesthetized on CO_2_ using a Flow Buddy CO_2_ regulator (Genesse Scientific, El Cajon, CA, USA). Microdissection tweezers and scissors were ethanol-sterilized before each replicate of dissections. Dissected abdomens were surface-sterilized in 70% ethanol and placed in a 1.5 mL microcentrifuge tube containing the DNeasy PowerSoil Pro Kit premade lysis buffer (QIAGEN, Germantown, MD, USA). Mosquito abdomens were homogenized using a mechanical homogenizer and sterile pestles for 2 min before proceeding with the manufacturer’s protocol.

### 2.4. DNA Amplicon Sequencing

DNA samples were sent to Molecular Research DNA Lab (MR. DNA) for amplification and sequencing. Amplicon sequencing using next-generation technology (bTEFAP^®^) was done as previously described and has been adapted to current NGS technologies [78]. The prokaryote 16S V4 primer pair, 515F GTGYCAGCMGCCGCGGTAA 806R GGACTACNVGGGTWTCTAAT, was utilized to evaluate the microbial ecology of each sample on the Illumina NovaSeq with methods via the bTEFAP^®^ DNA analysis service. Each sample underwent a single-step 35-cycle PCR using the HotStarTaq Plus Master Mix Kit (QIAGEN, Venlo, The Netherlands) under the following conditions: 95 °C for 5 min, followed by 30 cycles of 95 °C for 30 s, 53 °C for 40 s and 72 °C for 1 min, after which a final elongation step at 72 °C for 10 min was performed. Following PCR, all amplicon products from different samples were mixed in equal concentrations and purified using calibrated SPRI beads. Samples were sequenced utilizing the Illumina NovaSeq chemistry following the manufacturer’s protocols.

### 2.5. 16S rRNA Data Processing

The 16S rRNA sequencing data was processed using Trimmomatic to remove any adapter sequences. The remaining data was mapped to the Greengene dataset to identify taxonomy using the Kraken tools [79,80]. We used Kraken2 (version 2.1.3) to classify the sequences in the cleaned bacterial microbiota data to the Greengene dataset [81,82,83]. The bracken software (version 3.0.1) was then used to compute the abundance at the phylum, family, and genus levels from the samples using the Kraken2 output [84]. The overall distribution of bacterial families during different experimental conditions was plotted as bar charts using the ggplot2 package [85]. Total and classified read counts per sample; rarefaction curves; and phylum-, family-, and genus-level relative abundance for all samples are provided in Supplementary Data File S1.

### 2.6. Alpha Diversity

To compute the alpha diversity of the bacterial microbiota from different experimental diets before and after irradiation, the Shannon’s diversity index and the Berger–Parker (BP) index were used [80,81,82,83]. The Shannon diversity index was used to quantify biodiversity in each group by quantifying the number of bacterial phyla and their relative abundance. A high index represents more biodiversity of bacterial phyla in a group. The Berger–Parker (BP) index was used to measure the dominance of the most abundant bacterial phyla in each group. An index of 0 represents that no bacterial phylum has a dominant presence in the sample over other bacterial phyla. An index of closer to 1 represents that one bacterial phylum has more dominance in the sample over other bacterial phyla.

### 2.7. Beta Diversity

To compute the beta diversity between the bacterial microbiota from different experimental diets before and after irradiation, Python version 3.12.9 using the beta_diversity.py code provided with Kraken and Bracken tools was used to run a Bray–Curtis dissimilarity test [84,85,86]. The Bray–Curtis dissimilarity index was used to calculate how different each group is from others at the phyla level. An index of 0 represents no difference between the two groups, while an index of 1 represents a complete difference between the two groups. The metaMDS function of the vegan package was used to compute the non-metric multidimensional scaling (NMDS) plot. The NMDS was computed in two dimensions with multiple random states, to ensure convergence on a stable state.

### 2.8. Statistical Analysis

The results from the data analyses were statistically analyzed using GraphPad Prism Software (Version 11.0.0, San Diego, CA, USA) and Python (version 3.12.9, statsmodels package). The alpha diversity values of the BP index and Shannon’s diversity index were tested for normality using the Shapiro–Wilk test on the model residuals. Because larval diet and irradiation are two independent experimental factors, their individual and combined (interaction) effects were assessed using a two-way ANOVA, followed by Tukey’s HSD multiple comparison test. Residuals of the raw Shannon diversity index values deviated from normality (W = 0.852, *p* = 0.019). A square-root transformation was therefore applied prior to two-way ANOVA to satisfy the normality assumption (W = 0.928, *p* = 0.168 post-transformation). Residuals of the BP index met the normality assumption on the raw scale (W = 0.934, *p* = 0.313) and were analyzed untransformed.

## 3. Results

### 3.1. Bacterial Taxa from the Abdomens of Adult Male Ae. aegypti Reared on Different Larval Diets and Irradiation Statuses

**Figure 1:**
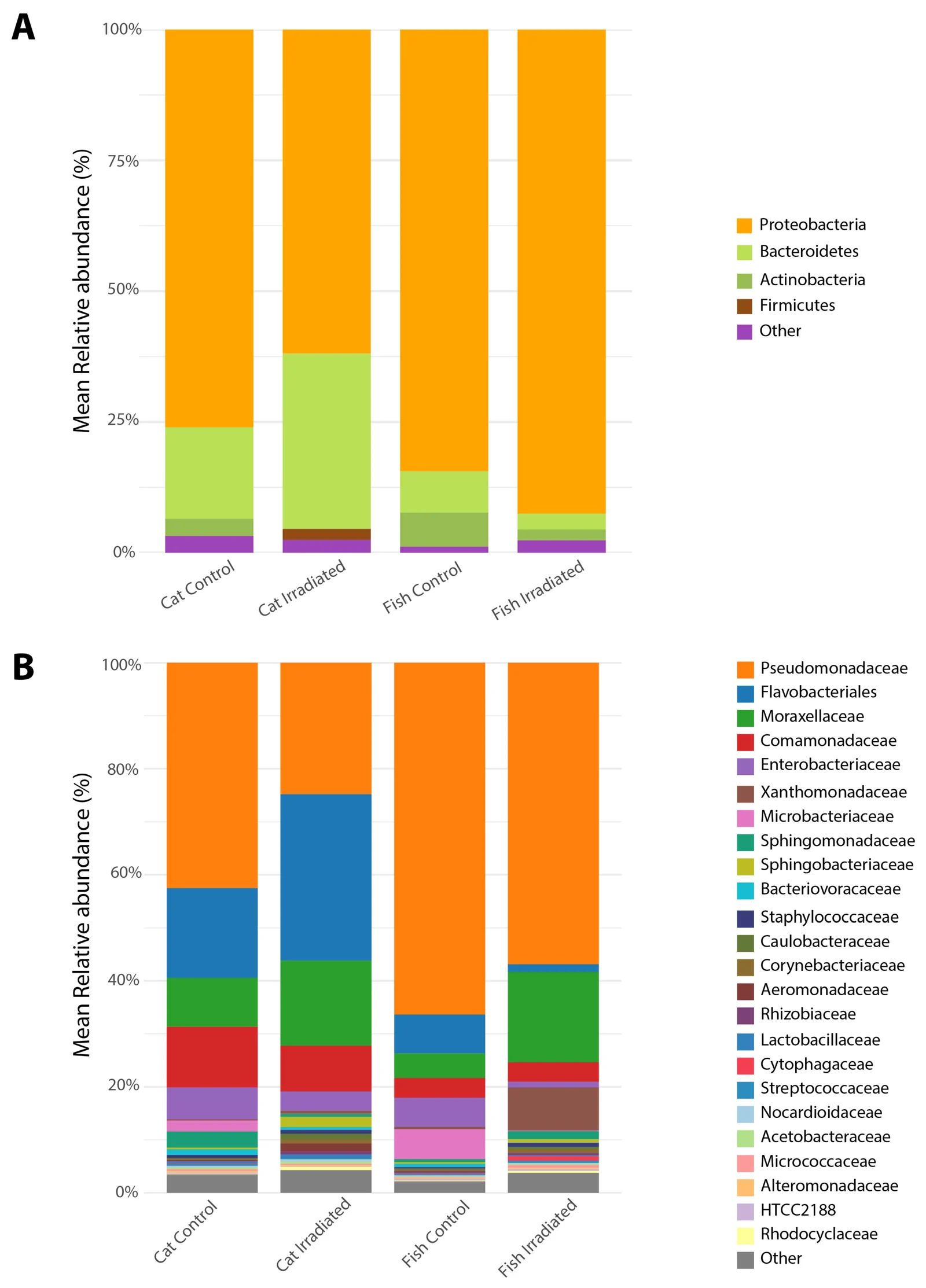
Larval diet and irradiation status impacts the relative abundance of bacterial phyla and families in adult male *Aedes aegypti* bacterial microbiota. Shown are the percent relative abundance of the bacterial (**A**) phyla and (**B**) families identified in *Ae. aegypti* abdomens that were reared on different larval diets (Cat or Fish) and were either not irradiated (control) or dosed with 50 Gy of ionizing radiation at the late pupal stage. Each column represents the bacterial phyla of four replicates of 50 mosquito abdomens each (total of 200 abdomens). (**A**) Phyla with less than 7% abundance are represented by the “Other” category in purple. (**B**) Families with less than 1% abundance are represented by the “Other” category in gray (bottom-most boxes).

In this study we found a total of 6,316,058 bacterial sequences from 16 grouped *Aedes aegypti* mosquito samples. The most abundant bacterial phyla in our samples were Proteobacteria (77.01%), Bacteroidetes (17.42%), Actinobacteria (3.39%), and Firmicutes (1.40%). Unclassified sequences accounted for less than 0.1% of the sequences. Another 21 phyla accounted for the remaining 1% of the sequences. Although the relative abundance of phyla changed across different diets, Proteobacteria was the most dominant bacteria present in male *Ae. aegypti* mosquito abdomens (see Figure 1A). The most abundant bacterial families in our samples were Pseudomonadaceae (47.79%), Flavobacteriales (16.25%), Moraxellaceae (10.83%), Comamonadaceae (6.25%), Enterobacteriaceae (4.11%), Xanthomonadaceae (2.17%), Microbacteriaceae (2.03%), and Sphingomonadaceae (1.30%). Again, the unclassified sequences accounted for less than 0.1% of the sequences. Another 168 families accounted for the remaining 9% of the bacterial communities. Although the relative abundance of bacterial families varied by diet and irradiation status, Pseudomonadaceae was the most abundant bacterial family present in the adult male *Ae. aegypti* abdomen for all samples except in the cat food irradiated group (see Figure 1B).

### 3.2. Larval Diet and Ionizing Radiation Treatment Impact Bacterial Richness and Diversity

**Figure 2:**
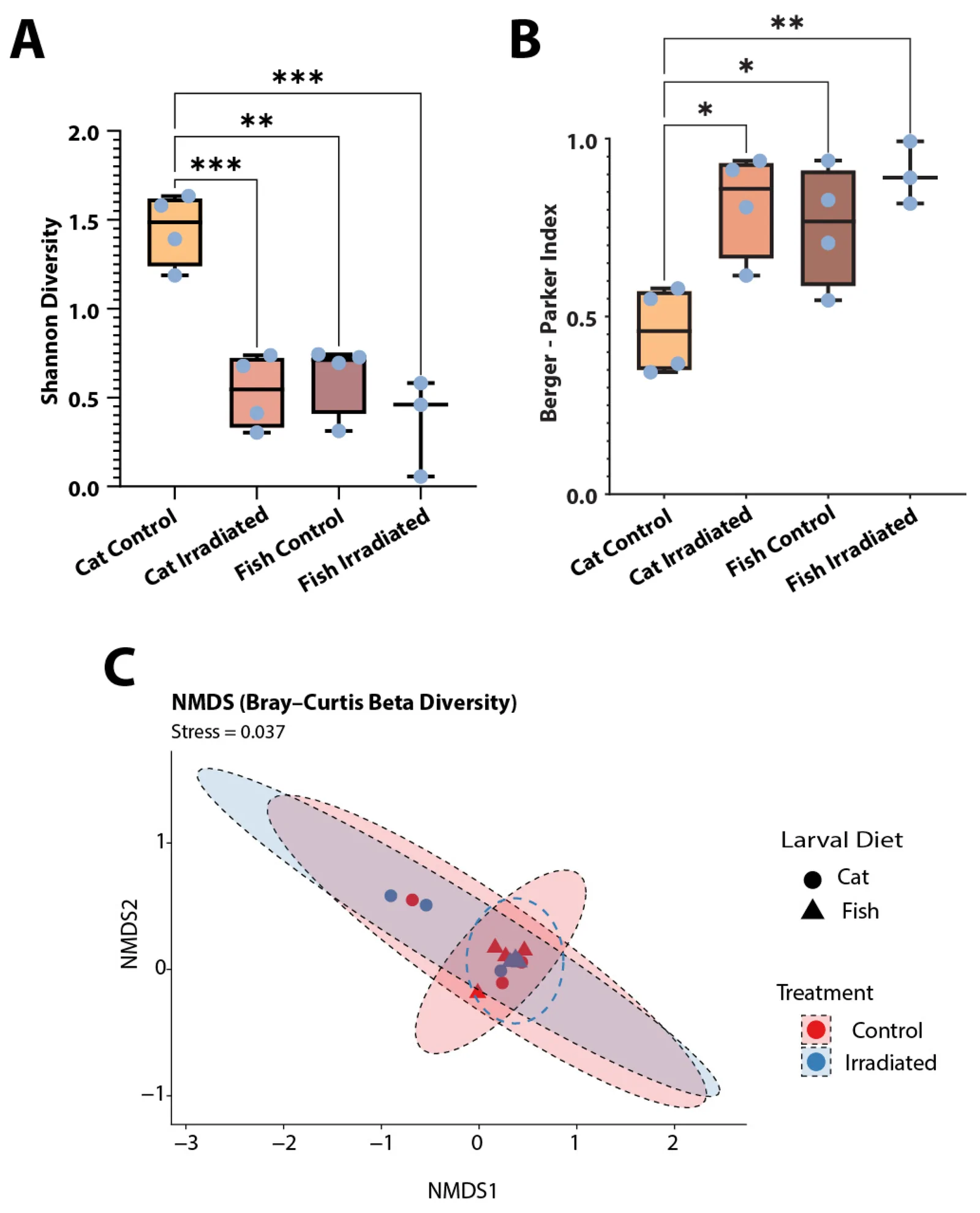
Larval diet and irradiation status impacts the adult male *Aedes aegypti* bacterial microbiota alpha and beta diversity. (**A**,**B**) Alpha diversity. Shown are box-and-whisker plots representing different indices used to measure alpha diversity in each treatment. The box-and-whisker plots show the median (center line), upper and lower quartiles of the data for each group. The whiskers represent the minimum and maximum values. Each gray dot represents a replicate. (**A**) The Shannon diversity index. ** Represents a *p*-value of 0.0012, and *** represents a *p*-value of <0.001. (**B**) The Berger–Parker (BP) index. * Represents a *p*-value of <0.05, and ** represents a *p*-value of 0.0071. (**C**) Beta diversity. Shown is an NMDS ordination plot that is using values from the Bray–Curtis dissimilarity metric. The filled-in circles and triangles represent mosquitoes that were reared on a cat or fish food larval diet, respectively. The ellipses denote the 95% confidence level for the distribution of each group. Red coloring represents control groups, and blue coloring represents the irradiated groups.

To assess the bacterial community diversity and richness of our different experimental groups, we measured the alpha and beta diversity. A two-way ANOVA showed that both larval diet (F(1,11) = 12.06, *p* = 0.0052) and irradiation (F(1,11) = 17.10, *p* = 0.0017) significantly reduced Shannon diversity, with no significant diet × irradiation interaction (F(1,11) = 2.29, *p* = 0.159). This indicates that diet and irradiation act independently on bacterial diversity. The average Shannon index for Cat Control and Cat Irradiated was 1.45 and 0.533, respectively. For Fish Control and Fish Irradiated, the Shannon index was 0.619 and 0.365, respectively. Tukey’s post hoc comparisons showed Cat Control had significantly higher bacterial diversity than all three other groups (all *p* < 0.02), which did not differ significantly from one another (see Figure 2A). For the BP index, a two-way ANOVA showed significant main effects of diet (F(1,11) = 7.55, *p* = 0.019) and irradiation (F(1,11) = 13.22, *p* = 0.0039), with no significant interaction (F(1,11) = 2.20, *p* = 0.166). The average BP index for Cat Control and Cat Irradiated was 0.460 and 0.818, respectively. In case of Fish Control and Fish Irradiated, it was 0.755 and 0.900, respectively. Tukey’s post hoc comparisons showed Cat Control had significantly lower bacterial dominance than all three other groups (all *p* < 0.05), which did not differ significantly from one another (see Figure 2B). The Bray–Curtis dissimilarity index values were plotted on an NMDS plot to visualize differences (see Figure 2C).

### 3.3. Larval Diet and Irradiation Status Shape Bacterial Genus-Level Composition

**Figure 3:**
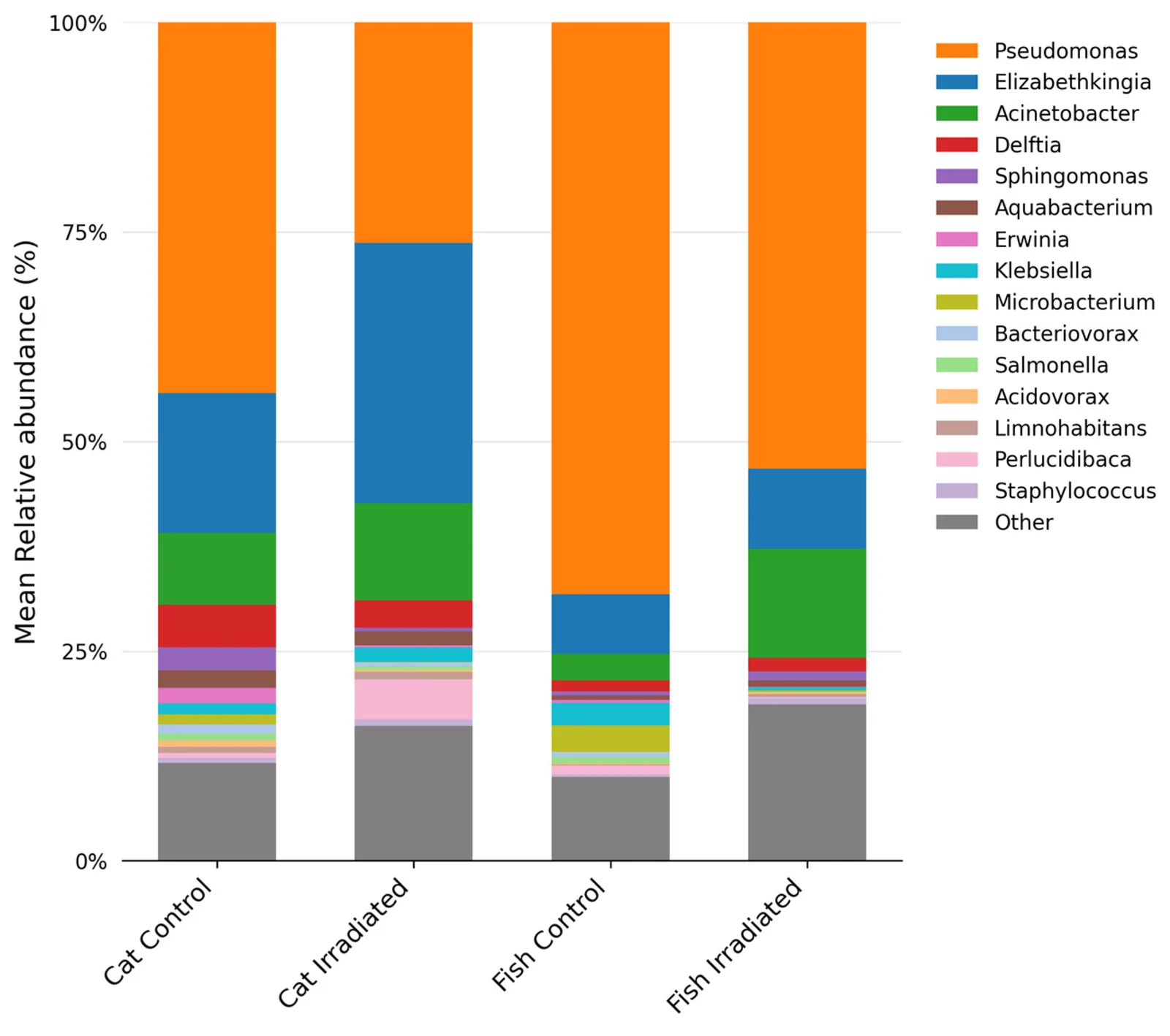
Larval diet and irradiation status impact bacterial genus-level composition in adult male *Aedes aegypti* bacterial microbiota. Stacked bar plots show the mean relative abundance (%) of the 15 most abundant bacterial genera in each treatment group, ranked by abundance in the Cat Control group. All remaining genera (382 total) are grouped as “Other” (gray). Genus-level relative abundance was computed from Kraken2/Bracken taxonomic classification of 16S rRNA amplicon sequences. Full genus-level data for all individual samples are provided in Supplementary Data File S1.

To address bacterial composition at finer taxonomic resolution, we examined the relative abundance of bacterial genera across the four treatment groups. *Pseudomonas* was the most abundant genus in all groups, ranging from 26.3% (Cat Irradiated) to 68.2% (Fish Control) of classified reads. *Elizabethkingia* was the second most abundant genus overall and was particularly enriched in the Cat Irradiated group (31.0%) relative to Cat Control (16.6%). Meanwhile, *Acinetobacter* was enriched in irradiated groups relative to their respective controls (Cat: 11.6% vs. 8.6%; Fish: 13.0% vs. 3.2%). The 15 most abundant genera accounted for 81.4–90.0% of classified reads per group, with the remaining genera (382 total) grouped as “Other” (see Figure 3).

### 3.4. Tetramin Fish Food and Special Kitty Cat Food Have Different Ingredients and Nutritional Content

**Figure 4:**
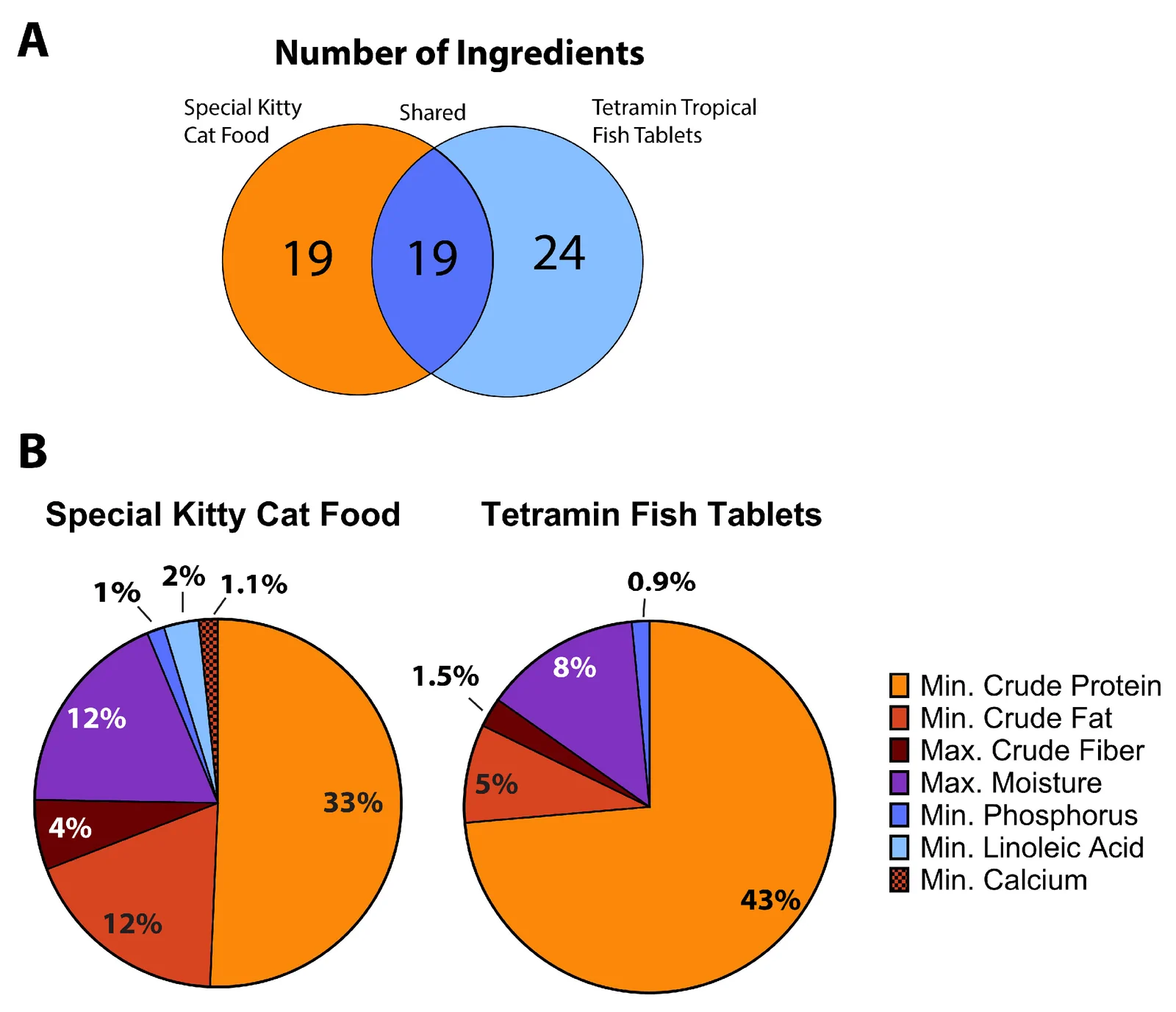
Composition of Tetramin fish food and the Special Kitty food diets. The two diets were compared using the ingredient lists and Guaranteed Analysis labels on both diet packages. (**A**) Venn diagram comparing the total number of shared and unique ingredients. The number inside each portion of the Venn diagram represents the number of ingredients. (**B**) Pie charts plotting the minimum and maximum crude nutrient content for both diets. The number within each slice of the pie chart represents the minimum or maximum weight percentage of crude nutrients claimed on the Guaranteed Analysis labels.

To assess the two diets tested in this study, we made a rudimentary comparison using their associated packaging ingredient lists and Guaranteed Analysis labels. Both diets were complex, with several ingredients and varying nutritional content. The Tetramin fish food and Special Kitty cat food had a total of 43 and 38 ingredients listed, respectively (see Supplemental File S1). The Special Kitty cat food shared half of its total ingredients with the Tetramin fish food (see Figure 4A). Both diets were also similar in caloric content with a disclosed, approximate label of 3513 kcalME/kg for the cat food and an approximately calculated 3650 kcalME/kg for the fish food. The Special Kitty cat food and Tetramin fish food, respectively, had a minimum crude protein content of 33% and 43%, minimum crude fat content of 12% and 5%, maximum crude fiber of 4% and 1.5%, maximum moisture content of 12% and 8%, minimum phosphorus content of 1% and 0.9%, minimum linoleic acid content of 2% and unknown, and a minimum calcium content of 1.1% and unknown (see Figure 4B).

## 4. Discussion

The mosquito microbiome has been investigated at the larval, pupal, and adult stages in several medically important mosquito vector species [86,87,88,89,90]. Overall, the microbiome, particularly of the midgut, has been shown to be important for larval molting and development, as well as adult longevity, reproduction, and vectorial capacity [73,74,75,76,77,91,92]. The microbiota composition in mosquitoes is influenced by a wide range of variables including environmental factors, diet, species, sex, and life stage [86,90,93,94,95]. There is strong evidence of transstadial transmission of some of the bacterial microbiota from larva to adult [90,96,97]. In addition, the supplementation of live bacteria to larval diets has been shown to improve growth and fitness in other Culicidae species and Mediterranean fruit flies [98,99].

Notably, there is nearly a complete absence of literature on the impact of irradiation on the mosquito microbiome. To the best of our knowledge Zhang and collaborators in 2021 were the first and only group to explore this topic [100]. In their study they investigated the effects of life stage, sex, and irradiation on *Aedes albopictus* bacterial gut microbiota. They found clear distinctions in the bacterial microbiota between all variables tested. Irradiation has been shown to affect the diversity and abundance of bacterial microbiota in other insects as well, including the Mediterranean and Oriental fruit flies and the tobacco cutworm moth [101,102,103]. In 2019, Asimakis and collaborators found that rearing the melon fly (*Zeugodacus cucurbitae*) on different larval diets resulted in different changes in the bacterial microbiota composition after irradiation [104]. Ami and collaborators in 2009 demonstrated that the addition of the bacterial species *Klebsiella oxytoca* to the diet of post-irradiated Mediterranean fruit flies significantly improved sterile male mating fitness [105].

Our current study on the adult male *Ae. aegypti* bacterial microbiota showed similar findings. Radiation treatment and larval diet both influenced the bacterial composition in male abdomens. It must be noted that the mosquito abdomen contains multiple tissues, including the midgut, fat body, testes and Malpighian tubules, which all may contain unique bacterial microbiota. We found that Proteobacteria, Bacteroidetes, Actinobacteria, and Firmicutes were the predominant phyla in male *Ae. aegypti* mosquito abdomens. These specific phyla have also been identified in *Ae. aegypti* and *Ae. albopictus* in several other microbiome studies [106,107,108,109]. Here we found that changes in the abundance of each of these phyla were associated with larval diet and irradiation status. Most notably, the relative abundance of Proteobacteria and Bacteroidetes showed opposite trends after irradiation in the cat food- and fish food-fed mosquitoes.

Mosquito larval diets used in various microbiome studies vary greatly, ranging from various in-house mixed yeast and liver powder recipes to commercially available aquatic animal diets like fish food and even other diets like cat food. Each of these diets have varying ingredients, nutritional contents, and costs. Cost is an important metric to consider for the mass-rearing of mosquitoes for population control methods like the SIT. Li and collaborators in 2025 tested the impact of three different larval diets on the development and fitness of *Ae. aegypti* [110]. Interestingly, they found that the cheapest diet tested, tortoise food, performed the best in *Ae. aegypti* compared with the other diets. This diet contained a nutritional composition of >38% protein, >4% fats, <8% dietary fiber, <4.5% calcium, and >1.5% total phosphorous. The other two diets were a 6:3:1 ratio of either pork liver powder or bovine liver powder, shrimp powder, and yeast powder. The bovine liver powder recipe was the most expensive diet tested in Li’s study. The nutritional composition of these diets containing either pork liver powder or bovine liver powder was 20% or >50% protein and 3.5% or <8% fats, respectively. These two diets had an unknown amount of fiber, calcium and total phosphorous. Li and collaborators attribute the success of the tortoise diet to the balance of macronutrients. In our study, we found that the cat food larval diet resulted in the highest bacterial diversity in adult male *Ae. aegypti*. This diet notably had a lower minimum crude protein content and a higher maximum crude fiber content than the fish food diet. Based on studies on the human gut microbiome, dietary fiber is the primary nutrient for maintaining gut microbiota diversity. Low-fiber diets have been associated with low microbiome diversity and negative health effects [111,112,113]. Considering our findings, Li’s findings, and the literature on human microbiomes, we suspect that fiber content and possibly calcium may directly influence the diversity of mosquito gut microbiota. Further analysis on the role of fiber in mosquito larval nutrition is needed.

Our future directions are to expand this study to incorporate probiotic supplements into larval diets and to include the current FOA/IAEA (Food and Agriculture Organization of the United Nations/International Atomic Energy Agency) larval diet recipe containing tuna meal, bovine liver powder, brewer’s yeast, squid powder, and a vitamin mix [114]. We will also follow-up our 16s rRNA results with mating, fitness, and survival assays. We will test the hypothesis that less change in bacterial microbiota before and after irradiation will result in fitter sterile males.

## 5. Conclusions

In conclusion, our hypothesis that specific larval diets will alter the effects of irradiation on the adult male *Aedes aegypti* bacterial microbiota is supported by the findings in this study. We also confirm that larval diet directly impacts adult bacterial microbiota. Our findings highlight an important gap in the literature on the impact of irradiation on mosquito bacterial microbiota and suggest that larval diet is a component to further study in the context of the SIT.

## Figures and Tables

**Table 1:** Naming key for different experimental groups analyzed in the study.

	Tetramin Fish Food Diet	Special Kitty Cat Food Diet
Not Irradiated (Control)	Fish Control	Cat Control
Irradiated (Treatment)	Fish Irradiated	Cat Irradiated

## Data Availability

All data generated and analyzed in this study are included in this published article and its Supplementary Materials. Raw 16S rRNA sequencing reads have been deposited in the NCBI Sequence Read Archive (SRA) under BioProject accession number PRJNA1469249.

## Supplementary Materials

The following supporting information can be downloaded at https://www.mdpi.com/article/10.3390/insects17090901/s1.
