# Supplementary material for: Sterile Insect Technique: Investigating the Impact of Larval Diet and Ionizing Radiation on the Mosquito Bacterial Microbiota
Source: Insects. 2026 Aug 27;17(9):901. doi: 10.3390/insects17090901 (PMC13607332; doi:10.3390/insects17090901)
Supplement: Supplementary file 1 [file insects-17-00901-s001.zip › File S1.pdf]

	Ingredients	Category
Shared	Fish oil	Fat source
	Soybean meal	Protein source
	Brewer’s yeast	Supplement
	Citric acid	Preservative
	Mixed tocopherols	Preservative
	Ferrous sulfate	Mineral
	Zinc sulfate	Mineral
	Niacin (B3)	Vitamin
	Riboflavin (B2)	Vitamin
	Thiamine (B1)	Vitamin
	Pyridoxine (B6)	Vitamin
	Vitamin B12	Vitamin
	Biotin	Vitamin
	Vitamin A	Vitamin
	Vitamin E	Vitamin
	Vitamin K (menadione)	Vitamin
	Calcium pantothenate	Vitamin
	FD&C Yellow #5	Colorant
	FD&C Blue #2	Colorant
Unique to Special Kitty Cat Food	**Chicken by-product meal**	**Protein source**
	**Ground whole grain corn**	**Carbohydrate**
	**Ground wheat**	**Carbohydrate**
	**Meat and bone meal**	**Protein source**
	**Animal fat (BHA preserved)**	**Fat source**
	**Natural flavor**	**Other**
	**Salt**	**Mineral**
	**Potassium chloride**	**Mineral**
	**Choline chloride**	**Supplement**
	**Taurine**	**Supplement**
	**DL-methionine**	**Supplement**
	**Zinc oxide**	**Mineral**
	**Copper sulfate**	**Mineral**
	**Manganous oxide**	**Mineral**
	**Sodium selenite**	**Mineral**
	**Folic acid**	**Vitamin**
	**Calcium iodate**	**Mineral**
	**Vitamin D3 supplement**	**Vitamin**
	**Cobalt carbonate**	**Mineral**

Unique to Tetramin Fish Tablets	Fish meal	Protein source
	Shrimp meal	Protein source
	Spirulina	Supplement
	Algae meal	Supplement
	Dried fish protein digest	Protein source
	Dried skimmed milk	Protein source
	Ground brown rice	Carbohydrate
	Potato protein	Protein source
	Feeding oatmeal	Carbohydrate
	Sucrose	Other
	Sorbitol	Other
	Lecithin	Other
	Monocalcium phosphate	Mineral
	Yeast extract	Supplement
	Rosemary extract	Preservative
	Propyl gallate	Preservative
	FD&C Red #3	Colorant
	Beta-carotene	Colorant/Nutrient
	Freshwater shrimp	Protein source
	Soybean oil	Fat source
	Wheat gluten	Protein source
	Manganese sulfate	Mineral
	Vitamin C	Vitamin
	Inositol	Supplement

Supplemental File S1. Ingredient comparison of the Special Kitty cat food and Tetramin fish food.
